# Neurological involvement in children with hemolytic uremic syndrome

**DOI:** 10.1007/s00431-021-04200-1

**Published:** 2021-08-10

**Authors:** Caoimhe Costigan, Tara Raftery, Anne G. Carroll, Dermot Wildes, Claire Reynolds, Robert Cunney, Niamh Dolan, Richard J. Drew, Bryan J. Lynch, Declan J. O’Rourke, Maria Stack, Clodagh Sweeney, Amre Shahwan, Eilish Twomey, Mary Waldron, Michael Riordan, Atif Awan, Kathleen M. Gorman

**Affiliations:** 1 Department of Nephrology, Children’s Health Ireland At Temple Street and Crumlin, Dublin, Ireland; 2 Department of Radiology, Children’s Health Ireland At Temple Street, Dublin, Ireland; 3 Department of Clinical Microbiology, Children’s Health Ireland At Temple Street, Dublin, Ireland; 4 Department of Neurology and Clinical Neurophysiology, Children’s Health Ireland At Temple Street, Dublin, Ireland; 5 School of Medicine and Medical Science, University College Dublin, Dublin, Ireland; 6 Department of Pediatrics, Royal College of Surgeons, Dublin, Ireland; 7 Irish Meningitis and Sepsis Reference Laboratory, Children’s Health Ireland At Temple Street, Dublin, Ireland; 8 Department of Clinical Microbiology, Royal College of Surgeons in Ireland, Dublin, Ireland; 9 Clinical Innovation Unit, Rotunda Hospital, Dublin, Ireland

**Keywords:** HUS, STEC-HUS, Neurology, Neurological involvement

## Abstract

Our objective was to establish the rate of neurological involvement in Shiga toxin-producing *Escherichia coli*–hemolytic uremic syndrome (STEC-HUS) and describe the clinical presentation, management and outcome. A retrospective chart review of children aged ≤ 16 years with STEC-HUS in Children’s Health Ireland from 2005 to 2018 was conducted. Laboratory confirmation of STEC infection was required for inclusion. Neurological involvement was defined as encephalopathy, focal neurological deficit, and/or seizure activity. Data on clinical presentation, management, and outcome were collected. We identified 240 children with HUS; 202 had confirmed STEC infection. Neurological involvement occurred in 22 (11%). The most common presentation was seizures (73%). In the *neurological group*, 19 (86%) were treated with plasma exchange and/or eculizumab. Of the 21 surviving children with neurological involvement, 19 (91%) achieved a complete neurological recovery. A higher proportion of children in the *neurological group* had renal sequelae (27% vs. 12%, *P* = .031). One patient died from multi-organ failure.

*Conclusion*: We have identified the rate of neurological involvement in a large cohort of children with STEC-HUS as 11%. Neurological involvement in STEC-HUS is associated with good long-term outcome (complete neurological recovery in 91%) and a low case-fatality rate (4.5%) in our cohort.

**Table:** 

**What is Known:** *• HUS is associated with neurological involvement in up to 30% of cases.* *• Neurological involvement has been reported as predictor of poor outcome, with associated increased morbidity and mortality.*	
**What is New:** *• The incidence of neurological involvement in STEC-HUS is 11%.* *• Neurological involvement is associated with predominantly good long-term outcome (90%) and a reduced case-fatality rate (4.5%) compared to older reports.*

**Supplementary information:**

The online version contains supplementary material available at 10.1007/s00431-021-04200-1.

## Introduction

Hemolytic uremic syndrome (HUS) is characterized by a triad of thrombocytopenia, microangiopathic hemolytic anemia, and kidney failure. Shiga toxin-producing *Escherichia coli* (STEC)-HUS is typically preceded by a diarrheal illness (usually bloody). In Ireland, the most common *Escherichia coli* (*E. coli*) serotype is O157:H7 [1]. Shiga toxin induces an inflammatory cascade, triggering endothelial injury, and thrombotic microangiopathy, resulting in micro-thrombi formation in multiple organs [2–4]. Activation of the alternative complement pathway may also have a role [5]. Shiga toxin-producing *E. coli*–HUS is the most common cause of acute kidney injury in children [1, 6–9]. Since 2004, STEC is a notifiable disease in Ireland and we consistently have the highest reported rate of STEC infection in Europe (19.4 per 100,000 in 2017) [10].

Neurological involvement is reported in approximately 30% of all types of HUS [2, 11–29]. Seizures, irritability, lethargy, encephalopathy, and coma are the most common central nervous system (CNS) manifestations. Neurological involvement in STEC-HUS is associated with a higher mortality rate (up to 30%), long-term physical disability, neuropsychological and cognitive sequelae (Supplementary Table 1) [11–16, 18, 21–25, 27–35].

Shiga toxin-producing *Escherichia coli* HUS is managed with careful fluid and diuretic administration, red cell transfusion, and in up to 50% of cases temporary kidney replacement therapy (continuous veno-venous hemofiltration (CVVH), peritoneal dialysis (PD), or hemodialysis (HD)) [36]. There is no consensus on the treatment of CNS involvement in STEC-HUS. Mixed outcomes have been reported after treatment with plasma exchange (PE) and/or anti-C5 monoclonal antibodies, e.g., eculizumab [13, 29, 34, 37–43].

We have reviewed all cases of STEC- HUS in children (≤ 16 years) referred to tertiary pediatric nephrology services in the Republic of Ireland over 13 years (*n* = 202). We report the rate of neurological involvement in this group, describe the clinical presentation, neurological and renal outcomes, and present an overview of management.

## Methods

### Study design

We undertook a retrospective chart review of children aged ≤ 16 years with STEC-HUS in Children’s Health Ireland, Dublin from, January 1, 2005, to December 31, 2018. Children’s Health Ireland is the sole provider of tertiary pediatric nephrology services in the Republic of Ireland. Patients were identified through the hospital discharge coding system and the nephrology patient database.

### Inclusion criteria

Children aged ≤ 16 years who met the clinical criteria for a diagnosis of HUS and laboratory confirmation of STEC infection were included.

### STEC-HUS case definition

Hemolytic uremic syndrome was defined as acute kidney injury, microangiopathic hemolytic anemia (hemoglobin < 10 g/dL with fragmented red cells), and thrombocytopenia (platelets < 150 × 10^9^/L). Laboratory confirmation of STEC was performed at the Health Service Executive Public Health Laboratory at Cherry Orchard Hospital, Dublin, which provides the National Reference service for STEC. Criteria for microbiological confirmation (as per national Health Surveillance and Protection Centre criteria) was as follows: (1) isolation of an *E. coli* strain by culture that is known to produce Shiga toxin (stx) or harbours *stx1* or *stx2* gene(s), or (2) direct detection of *stx1* or *stx2* nucleic acid (without strain isolation) by polymerase chain reaction (PCR), or (3) detection of *E. coli* serogroup specific antibodies [10].

### Neurological manifestations

Neurological involvement was defined as encephalopathy (altered or fluctuating level of consciousness), focal neurological deficit (abnormal neurological examination), and/or seizure activity. Patients were divided into those with neurological involvement (*neurological group)* and those without (*non-neurological group*). The *total group* refers to all children with STEC-HUS. Image interpretation was performed by two specialized pediatric radiologists.

### Exclusion criteria

Children with HUS who did not have confirmed STEC infection or had proven STEC infection but had a confirmed genetic diagnosis of atypical-HUS (aHUS) were excluded (Supplementary Fig. 1).

### Data collection

Data was collected from the medical and electronic charts regarding symptoms at presentation, laboratory values (urea, creatinine, hemoglobin, sodium, white cell count and differential at presentation and maximum values), microbiological results, investigations (radiology, electrophysiology), treatment, duration of stay, and long-term outcomes (defined below).

### Outcome

Renal sequelae were defined as the presence of one of the following: (1) hypertension requiring antihypertensive medication; (2) proteinuria (> 0.15 g/L or urinary protein-to-creatinine ratio greater than 20 mg/mmol); (3) impaired kidney function with an estimated glomerular filtration rate < 90 mL/min/1.73 m^2^ (Pediatric Schwartz formula) [44]. Neurological sequelae were defined as recurrent seizures, focal neurological deficit, or altered functional status at follow-up. The Pediatric Cerebral Performance Category (PCPC) was used as a qualitative assessment of overall neurological morbidity [45].

### Ethical approval

Ethical approval was granted by the local research and ethic committee.

### Statistical analysis

Data were analyzed using the SPSS version 26.0 (IBM SPSS Statistics, IBM Corporation). Frequencies and percentages were calculated to compare groups. To test for normal distribution, the Shapiro–Wilk test was used. For non-parametric data, quantitative and continuous variables were expressed as median and interquartile range (IQR). Non-parametric data were analyzed by Mann–Whitney *U* test and Pearson chi-square test. Statistical significance was determined at *P* value less than .05.

## Results

### Population

We identified 240 children with HUS. No evidence of STEC infection was detected in 36/240 (15%) patients. Two children had confirmed STEC infection but later developed recurrence of HUS in the absence of STEC infection—pathogenic complement gene mutations were subsequently identified. Two hundred two children with confirmed STEC infection (*total group*) were included in the analysis (Supplementary Fig. 1). Neurological involvement was identified in 22/202 children (11%) (Table 1).

**Table 1:** Demographics, clinical presentation, and management of pediatric HUS patients

	**Total HUS group, *n* = 202**	**HUS neurological group, *n* = 22**	**HUS non-neurological group, *n* = 180**	***P* value**
Demographics				
Gender, female:male, *n*	114: 88	13:9	101:79	.79
Age (years), median (IQR)	3.2 (1.6–6.3)	2.6 (1.1–7.4)	3.2 (1.6–6.2)	.44
Age category, *n* (%)				
< 1 year	15 (7.4)	4 (18)	11(6.1)	
≥ 1–2 years	44 (22)	4 (18)	40 (22)	.228
≥ 2–5 years	79 (39)	7 (32)	72 (40)	
≥ 5 years	64 (32)	7 (32)	57 (32)	
Season, *n* (%)				
Spring	35 (17)	4 (18)	31 (17)	
Summer	86 (43)	12 (55)	74 (41)	.381
Autumn	64 (32)	6 (27)	58 (32)	
Winter	17 (8.4)	0 (0.0)	17 (9.4)	
LOS, days				
Median (IQR)	10 (6.0–16.0)	21 (13.0–34.0)	9.0 (6.0–15.0)	< 0.001
ICU admission, *n* (%)	48 (24)	19 (86)	29 (16)	< 0.001
Presenting symptoms				
Incubation period, days				
Median (IQR)	(4.0–7.0)	5 (3.5–7.0)	5 (4.0–7.0)	.092
Diarrhea, *n* (%)	196 (97)	22 (100)	174 (97)	0.39
Urine output, *n* (%)				
Normal	45 (22)	1 (4.5)	44 (24)	
Anuria/oliguria	157 (78)	21 (95)	136 (76)	.034
Laboratory findings				
(admission) median (IQR)				
White cell count (× 10^9^/L)	13.2 (10.4–18.4)	16.1 (12–20)	13.0 (10.4–18.1)	.18
Neutrophils (× 10^9^/L)	7.0 (5.1–11.1)	7.4 (5.3–12.2)	6.8 (5.1–11)	.18
Hemoglobin (g/L)	87.0 (75–100.0)	87.5 (76.8–98.5)	87.0 (74.3–100.8)	.80
Platelets (× 10^9^/L)	51.0 (31–76)	51.5 (29.8–63.5)	50.0 (31–78)	.82
Urea (mmol/L)	23.4 (15.2–32.1)	24.6 (16.1–37.1)	23.3 (15.1–32)	.63
Creatinine (μmol/L)	211 (121.5–314.5)	297.5 (148.5–371.3)	208 (120–305)	.06
Max. creatinine (μmol/L)	349.5 (150.5–578.8)	383 (271.5–737.5)	349 (142–576)	.82
Sodium (mmol/L)	134 (132–136)	135 (132–138)	134 (132–136)	.34
Treatment				
Dialysis, *n* (%)	107 (53)	19 (86)	88 (49)	< .001
CVVH	16 (7.9)	6 (27)	10 (5.6)	
PD	83 (41)	10 (46)	73 (41)	.016
CVVH and PD	8 (4.0)	3 (14)	5 (2.8)	
Duration, days, median (IQR)	9 (6–13)	11 (7.3–19)	9 (6–13)	.81
PE, *n* (%)	24 (12)	15 (68)	9 (5.0)	
Duration, days, median (IQR)	4 (2.5–5)	4 (3–55)	4 (1–5)	< .001
Eculizumab, *n* (%)	8 (4.0)	8 (36)	0 (0)	0.503

No missing data

*CVVH* continuous veno-venous hemofiltration, *HUS* hemolytic uremic syndrome, *ICU* intensive care unit, *IQR* interquartile range, *LOS* length of stay, *max* maximum, *n* number, *PD* peritoneal dialysis, *PE* plasma exchange

### Microbiology

A Shiga toxigenic strain of *E. coli* was isolated in 187/202 (92%) children on stool culture; 172 also had *stx* detected on PCR and 37 had antibodies on serology. Seven *E. coli* serotypes were identified; O157 (50%) and O26 (30%) were the most common. Sixteen patients (8%) had an ungroupable *E. coli* serotype (Table 2). In 4 patients, *stx* was detected without isolation of an *E. coli* strain. Both *stx1* and *stx2* were detected in 43 children, *stx2* alone in 109 and *stx1* alone in three. Unspecified *stx* was identified in 18 and no *stx* detected in 27.

**Table 2:** *E. coli* serogroups identified from pediatric HUS patients

***Escherichia coli***	**Total HUS group, *n* = 202 (%)**	***stx* detected, *n* (%)**	**Neurological group, *n = 22***	***stx* detected, *n* (%)**	**Non-neurological group, *n* = 180 (%)**	***stx* detected, *n* (%)**	*P* value^*^
*E. coli* O157, *n* (%)	101 (50)	86 (85)	7 (29)	5(66)	94 (52)	81 (86)	0.05
*E. coli* O26, *n* (%)	62 (30)	53 (85)	8 (38)	6 (75)	54 (30)	47 (87)	0.34
Ungroupable, *n* (%)	16 (7.9)	12 (75)	4 (19)	3 (75)	12 (6.6)	9 (75)	0.05
*E. coli* O145, *n* (%)	13 (6.4)	12 (92)	2 (9.5)	2 (100)	11 (6)	10 (83)	0.20
*E. coli* O103, *n* (%)	4 (2.0)	4 (100)	0	0	4 (2.2)	4 (100)	0.49
Shigatoxin only, *n* (%)	4 (1.5)	4 (1.5)	0	0	4 (2.2)	4 (2.2)	0.49
*E. coli* O111, *n* (%)	3 (1.5)	3 (100)	1 (4.8)	1 (100)	2 (1.1)	2 (100)	0.19
*E. coli* O132, *n* (%)	1 (0.5)	1 (100)	0	-	1 (0.6)	1 (100)	0.73
*E. coli* O78, *n* (%)	1 (0.5)	1 (100)	0	-	1 (0.5)	1 (100)	0.80
Total	205^**^	176^**^ (85)	22	16 (76)	183^**^	160^**^ (86)	**-**

*HUS* hemolytic uremic syndrome, *PCR* polymerase chain reaction, *stx* shigaotoxin

^*^Difference in serotypes between neurological and non-neurological group; ^**^Three patients had both O26 and O157

### Clinical presentation

At presentation, 196/202 (97%) children had diarrhea, of whom 121/196 (61%) had bloody diarrhea, and 45/202 (22%) were febrile. There was a significantly higher proportion of patients in the *neurological grou*p with oliguria or anuria (96% vs. 76% (*P* = .034)). The degree of leukocytosis, thrombocytopenia, anemia, and hyponatremia at presentation were not significantly different between groups. Admission and peak creatinine were similar in both groups (Table 1). Admission rate to the PICU was higher [86% vs. 16% (*P* < .001)] and median length of hospital stay was longer [21.0 days vs. 9.0 days (*P* < .001)] in the *neurological group.*

### Neurological presentation

The *neurological group* comprised of 22 children (Table 3). The median time from admission to the onset of CNS symptoms (seizures, encephalopathy or focal neurological impairment) was one day (IQR 0.0–2.3 days). Seizure was the most common presentation [16/22 (73%)]; four children presented with status epilepticus. Ten patients were clinically encephalopathic, and four had focal neurological deficits. Anti-epileptic medications were used during hospital admission in 16 patients; four remained on medication at discharge. All medications had been discontinued by 6 months post-discharge.

**Table 3:** Presentation, investigations, and outcome of the neurological cohort (*n* = 22)

**No**	**CNS symptoms**	**Acute radiological features of HUS**		**EEG**	**Treatment**	**AEDs**	**Neurological outcome (PCPC score)**
		**CT**	**MRI**				
1	Encephalopathic	-	RD and T2-hyperintensity in the centrum semi-ovale and PVWM	-	PE	BZD	Normal (1)
	GTCS		*Follow-Up*: Resolved			*Discharge:* Nil	
2	Encephalopathic	-	-	Absence of cerebral activity	PE then Eculizumab	-	Deceased
	Hypotonic						
3	Encephalopathic	-	-	-	Eculizumab	-	Normal (1)
4	GTCS	-	-	-	-	BZD	Baseline (2)
						*Discharge:* Nil	
5	Status Epilepticus	No	-	-	PE	BZD, PHY	Normal (1)
						*Discharge:* Nil	
6	Seizure	No	No	Slow	PE	VPA	Baseline (2)
						*Discharge:* VPA	
7	Encephalopathic	No	No	Slow	Eculizumab	BZD, PHY, LEV	Normal (1)
	GTCS then prolonged focal seizure later in admission					*Discharge:* LEV	
8	GTCS	-		-	Eculizumab	PHY	Normal (1)
						*Discharge*: Nil	
9	Encephalopathic	-	No	-	PE	BZD	Normal (1)
	GTCS					*Discharge:* Nil	
10	GTCS	No	No	Slow	Eculizumab	-	Normal (1)
11	Encephalopathic	-	No	-	PE	BZD, PHB, PHY	Normal (1)
	Status epilepticus					*Discharge:* Nil	
12	GTCS	No	-	-	PE	BZD	Normal (1)
						*Discharge*: Nil	
13	Encephalopathic	No	-	Slow	PE	-	Normal (1)
14	Status epilepticus	Low attenuation in bilateral BG and THAL	T2 hyperintensity and mixed increased/RD in BG and THAL	-	PE then eculizumab	PHB, PHY, THI	Normal (1)
	Focal seizures		*Follow-Up*: Improved			*Discharge:* PHY	
15	Encephalopathic Hemiparesis GTCS	Loss of GWM differentiation in R occipital lobe	T2 and FLAIR hyperintensity in the PVWM bilaterally (R > L) and R occipital lobe	Abnormal	PE	PHB, PHY *Discharge:* PHB	Normal (1)
16	Prolonged focal motor seizure Encephalopathic Abnormal tone	Low attenuation in bilateral BG and THAL	T2 hyperintensity and mixed increased/RD in BG and THAL	Slow	Eculizumab then PE	BZD *Discharge:* Nil	Mild Impairment (2) Difficulty with complex motor tasks
17	Encephalopathic	-	-	Slow	PE	LEV *Discharge:* Nil	Normal (1)
18	Status epilepticus	-	RD in WM and BG *Follow-Up:* Resolved	Slow	PE then eculizumab	BZD, PHB *Discharge:* Nil	Normal (1)
19	Dysarthria and weakness	-	-	-	-	-	Mild Impairment (2) Dysarthria, mild weakness
20	Focal motor seizure	-	RD in centrum semi-ovale and PVWM T2 hyperintensity in centrum semi-ovale	Slow	PE	BZD, LEV *Discharge*: Nil	Normal (1)
21	Left 4th CN palsy L upper limb weakness	No	Increased DWI and edema in cerebellum	-	PE	-	Normal (1)
22	GTCS	No	-	-	-	BZD *Discharge:* Nil	Normal (1)

*AED* antiepileptic drugs, *BG* basal ganglia, *BZD* benzodiazepine, *CN* cranial nerve, *CNS* central nervous system, *CT* computed tomography, *DWI* diffusion-weighted imaging, *EEG* electroencephalogram, *GTCS* generalized tonic–clonic seizure, *L* left, *LEV* levetiracetam, *MRI* magnetic resonance imaging, *PCPC* Pediatric Cerebral Performance Category, *PE* plasma exchange, *PHB* phenobarbitone, *PHY* phenytoin, *PVWM* periventricular white matter, *R* right, *RD* restricted diffusion

Electroencephalogram was performed in 10 children during the acute illness. All studies were abnormal, with a slow background consistent with encephalopathy in nine and absent cerebral activity in one.

**Fig. 1:**
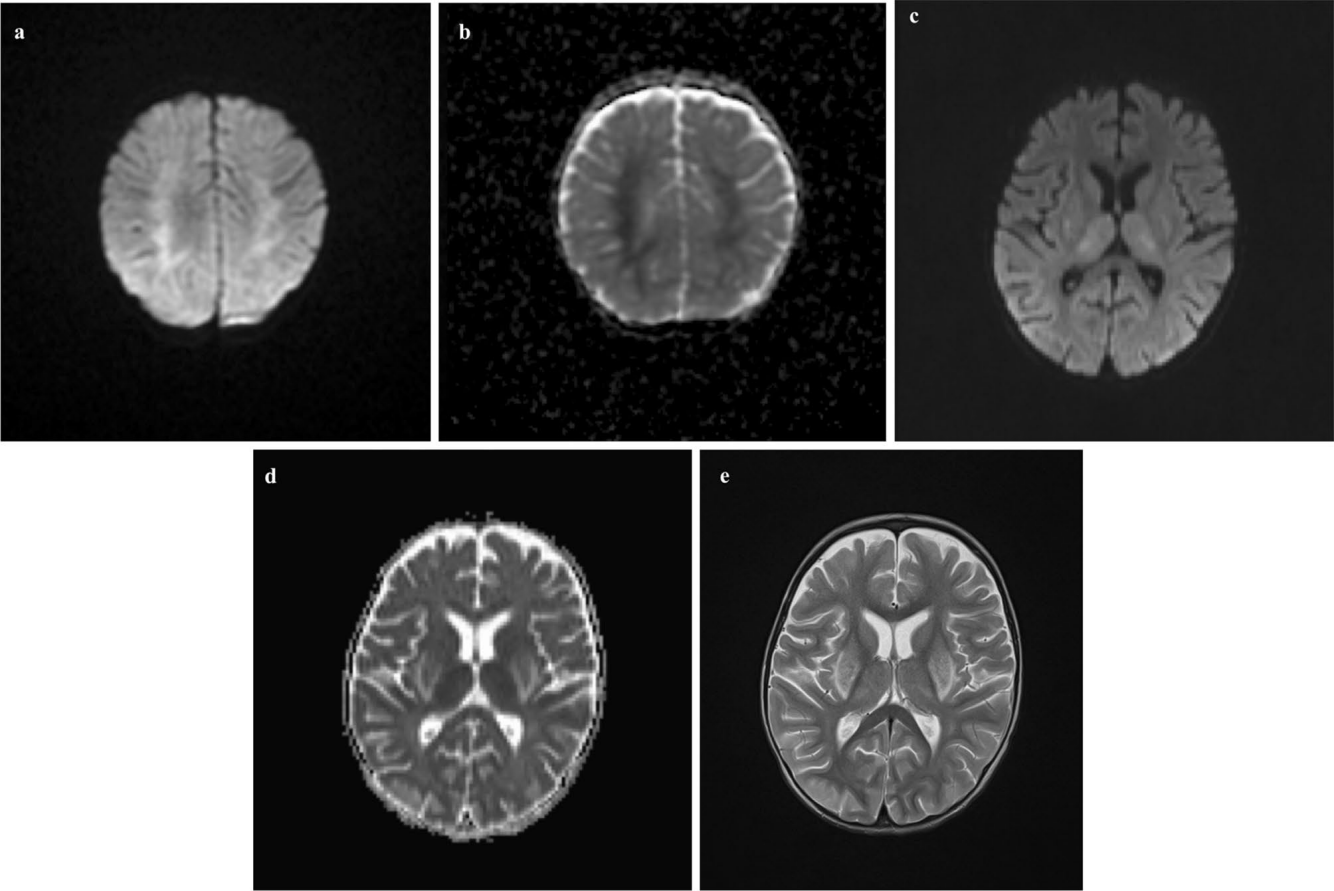
**a**, **b** Patient 1: axial DWI (**a**) and ADC map (**b**) show reduced diffusivity in both centrum semi-ovale which extended inferiorly to the periventricular white matter adjacent to bilateral frontal horns. **c–e** Patient 16: axial DWI (**c**) and ADC map (**d**) show restricted diffusion in the thalami bilaterally with increased diffusion within the periphery of the lentiform nuclei. There is corresponding increased signal abnormality in grey matter structures on axial T2 images (**e**)

Neuroimaging was available in 17 children (Table 3). Ten patients had no acute findings on neuroimaging despite clinical evidence of encephalopathy (3 magnetic resonance imaging (MRI); 4 computer tomography (CT); 3 both MRI and CT). There was no difference in the timing of imaging between those with or without acute changes. Table 3 summarizes neuroimaging findings. Figure 1 illustrates the typical restricted diffusion pattern on diffusion-weighted imaging (DWI). Three patients had follow-up MRI studies which showed improvement or complete resolution.

### Management

In the *total group*, 107/202 (53%) required dialysis. Significantly more patients in the *neurological group* needed dialysis than in the *non-neurological group* (86% vs. 49%, (*P* < .001)). The most common modality utilized was PD in 83/107 (77%); CVVH in 16 (15%) and 8 (7.5%) children had both.

In the *total group*, 24/202 (12%) had PE; 15/22 (68%) in the *neurological group* and 9/180 (5.0%) in the *non-neurological group*. The median number of PE sessions in the *neurological group* was 4.0 (IQR 3.0–5.0). Patients who received PE without evidence of CNS involvement (*n* = 9) did so due to atypical presentation before the confirmation of STEC. Eight children had eculizumab, all in the *neurological group* (Supplementary Fig. 1)*.*

In the *neurological group*, 19/22 patients (86%) had either PE or eculizumab (Table 3). Three patients received neither—one had a single seizure in a referring hospital but was not clinically encephalopathic on arrival at our center (*patient 4*); one had a prolonged PICU admission and neurological deficits were only noted post-extubation (*patient 19*); one had a seizure thought to be related to severe hypertension at the time **(***patient 22*). One patient received eculizumab then PE (4 days later) due to the re-emergence of neurological signs (abnormal neurological examination, increased tone and altered level of consciousness) despite an initial improvement (*patient 16*). Three received PE then eculizumab—due to concerns regarding response to the initial treatment or evolving diagnostic uncertainty. Plasma exchange was commenced within 24 h of the onset of neurological symptoms in 13 of 15 (87%) cases. Four patients were treated with eculizumab alone. All patients who received eculizumab initially did so within 24 h.

One patient in the *neurological group* died from multi-organ failure. Two patients developed central line related deep venous thrombosis requiring anticoagulation. One of these patients also received treatment for an associated fungal infection. All patients who got eculizumab were given antibiotic prophylaxis and appropriate meningococcal vaccination.

### Outcomes

#### Neurological

Of the 21 surviving patients with neurological involvement, 19/21 (91%) made a complete recovery. Two patients (9.5%) had mild impairment on PCPC at both discharge and most recent follow-up: both reported difficulties with complex motor tasks. One patient developed a brief generalized onset-motor seizure 1-year post-discharge but was not commenced on anti-epileptic medication.

#### Renal

Complete follow-up data were available on 178/202 children (88%), 18 (9%) were referred to regional pediatric centers for follow-up, and 5 (2.5%) were lost to follow-up. Renal recovery was achieved in 154/178 (87%) after a median follow-up of 2.4 years (IQR 0.7–5.5 years). A greater proportion of patients in the *neurological group* had renal sequelae (27% vs. 12%; *P* 0.031) (Table 4). Two patients, one from each group, developed stage 5 chronic kidney disease and were transplanted.

**Table 4:** Renal outcome of the total group with STEC-HUS

	**Total group, *n* = 202 (%)**	**Neurological group, *n* = 22 (%)**	**Non-neurological group, *n* = 180 (%)**	***P* value**
Long-term data available	178 (88)	21 (96)	157 (87)	
Regional center follow-up	18 (8.9)	0	18 (10)	.11
Lost to follow-up	5 (2.5)	0	5 (2.8)	
Deceased	1 (0.5)	1 (4.5)	0	
	**Total group, *n* = 178 (%)**	**Neurological group, *n* = 21 (%)**	**Non-neurological group, *n* = 157 (%)**	
Duration of follow-up, years, median (IQR)	2.4 (0.7–5.5)	3.6 (2.3–4.6)	2.2 (0.6–5.6)	.037
Complete renal recovery	154 (87)	15 (71)	139 (89)	.031
Long-term renal sequelae	24 (14)	6 (27)	18 (12)	.031
Proteinuria	14 (7.9)	5 (24)	9 (5.7)	.004
Hypertension	7 (3.9)	1 (4.8)	5 (3.2)	.707
Mild impairment (eGFR 60–89 mL/min/1.73 m^2^)	6 (3.4)	3 (14)	3 (1.9)	.003
Mild/moderate impairment (eGFR 45–59 mL/min/1.73 m^2^)	4 (2.2)	0	4 (2.5)	.459
CKD 5-transplant	2 (1.1)	1 (4.8)	1 (0.6)	`.092

Definitions: complete renal recovery, absence of proteinuria or hypertension and a normal eGFR; hypertension, ≥ 95th percentile for age, height, and sex and requiring an antihypertensive medication; proteinuria, > 0.15 g/L or urinary protein-to-creatinine ratio greater than 20 mg/mmol

*eGFR* estimated glomerular filtration rate, *CKD* chronic kidney disease, *n* number

## Discussion

We have identified that the rate of neurological involvement in STEC-HUS is 11%. Neurological involvement is associated with predominantly good long-term outcome (90%) and a reduced case-fatality rate (4.5%) compared to older reports.

The reported rate of neurological involvement in children with HUS varies between 10 and 52% (Supplementary Table 1) [11–16, 18, 20–25, 27–35]. We report a rate of 11% based on a strict definition of neurological involvement (seizures, encephalopathy or focal neurological deficit). We considered features such as irritability or lethargy to be non-specific. Four patients met the criteria for neurological involvement, but STEC infection was not confirmed, and they were excluded; no alternative etiology was identified. The possibility of failure to identify STEC infection in this small group exists—their inclusion would increase the rate of neurological involvement to 12%.

Seizure (16/22 (73%)) was the most common presentation of neurological involvement*.* Neurological involvement was noted early in the disease course, manifesting within 48 h of admission to hospital in 73%. Close monitoring of CNS symptoms and careful clinical assessment is important to identify neurological involvement in children with HUS early in the course of their disease.

Demographic variables did not predict neurological involvement in our cohort, and we did not identify a trend for a higher degree of leukocytosis or peak creatinine, as reported previously (Table 1) [14, 18, 46]. The *E. coli* serogroups identified were comparable between the *neurological* and *non-neurological* groups. Children in the *neurological group* had a significantly greater need for dialysis (86% vs. 49%, (*P* < .001)), PICU admission (86% vs. 16%, (*P* < 0.001)), and a longer length of hospital stay (21 (13–34) days vs. 9 (6–15) days, (*P* < 0.001)), reflecting a more severe course of illness in this group. The implementation of a HUS prognostic index score has been proposed as a predictor of short and long-term outcomes [47]. However, in our cohort, it did not predict CNS involvement, length of stay, mortality, or long-term sequelae (Supplementary Table 3). Therefore, other markers of disease severity or prediction score needed to be developed to predict disease severity.

Neuro-radiology in children with HUS is focused on the exclusion of hemorrhage and the identification of cerebral edema and vasculitis [19]. Children in the *neurological group* underwent CT and/or MRI depending on their individual clinical circumstances. Based on availability, and if patients are sufficiently stable to allow for a longer examination duration, MRI is the imaging modality of choice. We identified the typical DWI abnormalities of both deep white and grey matter in our patients (*n* = 7) [13]. All children who had DWI changes had a normal neurological outcome (Table 3). Therefore, observed DWI changes are reversible lesions in children with good neurological recovery and routine follow-up neuroimaging is not required, unless abnormal neurological examination.

Evidence supporting the use of supplemental treatments, such as PE or eculizumab, in STEC-HUS is lacking [48–51]. Extensive cases series and small cohort studies have been published but no randomized control trials have been reported (Supplementary Table 2) [13, 15, 29, 33, 34, 37–43, 52–54]. Many specialists, while cautiously skeptical of the role of such treatments, tend to use supplemental therapies in severe cases of HUS, particularly in the context of CNS involvement [48–51]. In our cohort, we reserved additional treatments for children with severe disease, treating 20/202 children (9.9%) with PE, 4/202 (1.9%) with eculizumab, and 4/202 (1.9%) with both. Our initial approach is treatment with PE; reserving Eculizumab for use when prompt initiation of PE is not practicable or if overwhelming multi-system involvement. It is important to avoid the simultaneous use of PE and eculizumab as monoclonal antibodies will be removed by PE. It is difficult, based on our positive experience, to forgo supplemental therapies until the outcomes of randomized controlled trials are available.

Neurological involvement in HUS has been reported to be associated with high mortality and significant long-term neurological morbidity (Supplementary Table 1) [11–16, 18, 20–25, 27–35]. Reported outcomes vary depending on the period studied and the case definition employed. Studies based on cohorts of children with HUS and CNS involvement before 2010 had a median mortality rate of 17% (IQR 7.0–45%) [11, 12, 19, 21–25, 27, 28, 30] and long-term neurological sequelae of 14% (IQR 11–33%) [12, 19, 22, 24, 30, 31]. More recent studies (cohorts after 2010) have better outcomes with lower mortality (14%, (IQR 13–22%)) [13, 14, 16, 18, 20, 34] and less long-term neurological sequelae (8.3%, IQR 5.3–35%)) [13, 15, 16, 18, 33–35]. Substantial improvements in diagnosis and supportive care have evolved in the intervening period. In our *neurological group* (*n* = 22), one patient died, and two children had long-term neurological consequences—giving comparative rates of 4.5% case-fatality, 9.5% mild neurological sequelae, and no severe neurological sequelae. The rate of kidney sequelae on follow-up is significantly better than that described in other cohorts—14% vs 20–25% [2, 54]. It is likely that advances in supportive care are the primary driver for improved outcomes (renal and neurological) in our cohort compared to older published cohorts. We believe that more optimism should be afforded when counseling parents regarding long-term neurological and renal sequelae.

Making comparisons between cohorts of HUS patients treated using different protocols in different centers is not optimal. Differences in the case definition, case capture, inclusion of aHUS patients, length of follow-up, and treatment modalities, along with demographic variables and the genetic background of the population make comparisons complex. Our cohort benefits from a high degree of case capture based on a well-defined geographical area served by a single tertiary center—near complete capture of HUS cases with more mild disease involvement will have an impact on measurement of overall disease severity.

One important limitation of our study is the ability to detect more subtle changes in neurocognitive function and behavior. The PCPC was developed to quantify overall functional morbidity in children after critical illness—application of more sensitive scales may allow for the detection of more subtle changes in neurocognitive outcomes and behavior [46, 55–57].

## Conclusion

One in ten children with STEC-HUS will have neurological involvement and 90% will have a complete neurological recovery. The optimal management of neurological involvement in STEC-HUS needs further study. In the absence of good quality randomized control studies, it is important that cohort studies are reported.

## Supplementary information

Below is the link to the electronic supplementary material.

Supplementary file1 (DOCX 21 KB)

Supplementary file2 (DOCX 21 KB)

Supplementary file3 (DOCX 18 KB)

Supplementary file4 (JPEG 149 KB)

## Data Availability

Data can be provided on request.

## Abbreviations

aHUS: Atypical-HUS
CNS: Central nervous system
CT: Computed tomography
CVVH: Continuous veno-venous hemofiltration
DWI: Diffusion-weighted imaging
HD: Hemodialysis
HSPC: Health Surveillance and Protection Centre
HUS: Hemolytic uremic syndrome
IQR: Interquartile range
MRI: Magnetic resonance imaging
PCPC: Pediatric Cerebral Performance Category
PCR: Polymerase chain reaction
PD: Peritoneal dialysis
PE: Plasma exchange
PICU: Pediatric intensive care unit
STEC: Shigatoxin-producing *Escherichia coli*
stx: Shigatoxin
