# Supplementary material for: Neurological involvement in children with hemolytic uremic syndrome
Source: Eur J Pediatr. 2021 Aug 10;181(2):501–12. doi: 10.1007/s00431-021-04200-1 (PMC8821508; doi:10.1007/s00431-021-04200-1)
Supplement: Supplementary file 1 — Supplementary file1 (DOCX 21 KB) [file 431_2021_4200_MOESM1_ESM.docx]

| Supplementary Table 1: Summary of available literature on neurological involvement in HUS | | | | | | | |
| --- | --- | --- | --- | --- | --- | --- | --- |
| Study | Year of  Publication | Years  Studied | Children/  Adults | *N* | STEC | Neurological  Involvement | % |
| Rooney et al. | 1971 | - | C | 25 | - | 13 | 52 |
| Bale et al. | 1980 | 1972-1978 | C | 61 | - | 30 | 49 |
| Sheth et al. | 1986 | - | C | 44 |  | 15 | 34 |
| Hahn et al. | 1989 | 1976-1986 | C | 78 | - | 16 | 21 |
| Martin et al. | 1990 | 1979-1988 | C | 117 | 13 | 18 | 15 |
| Cimolai et al. | 1992 | 1982-1990 | C | 91 | - | 27 | 30 |
| Siegler et al. | 1994 | 1971-1990 | C | 157 | 47 | 44 | 28 |
| Eriksson et al. | 2001 | 1985-1992 | C | 22 | 3 | 22 | 100 |
| Banatavala et al. | 2001 | - | 73 C | 83 | - | 25 | 30 |
| Gerber et al. | 2002 | 1997-2000 | C | 344 | 327 | 87 | 25 |
| Steinborn et al. | 2004 | 1995-2003 | C | 57 | - | 17 | 30 |
| Nathanson et al. | 2010 | 1975-2008 | C | 52 | 24 | 52 | 25 |
| Loos et al. | 2012 | - | C | 90 | 90 | 23 | 26 |
| Rosales et al. | 2012 | 1997-2002 | C | 619 | 489 | 130 | 21 |
| Gitaux et al. | 2013 | 2010-2011 | C | 14 | 14 | 9 | 64 |
| Pape et al. | 2015 | 2011-2014 | C | 11 | 11 | 11 | 100 |
| Matthies et al. | 2016 | 2004-2015 | C | 46 | 40 | 8 | 17 |
| Loos et al. | 2017 | 2011 | C | 72 | 72 | 19 | 26 |
| Tavasoli et al. | 2019 | 2001-2015 | C | 58 | - | 12 | 21 |
| Giordano et al. | 2019 | 2006-2016 | C | 54 | 54 | 12 | 22 |
| Ylinen et al. | 2020 | 200-2016 | C | 87 | 58 | 29 | 33 |
| Brown et al. | 2021 | 2004-2018 | C | 3915 | - | 409 | 10.4 |

Abbreviations: C; children, N; total number in study, STEC; shiga toxin prodcuing *E.coli.*
