# Supplementary material for: Neurological involvement in children with hemolytic uremic syndrome
Source: Eur J Pediatr. 2021 Aug 10;181(2):501–12. doi: 10.1007/s00431-021-04200-1 (PMC8821508; doi:10.1007/s00431-021-04200-1)
Supplement: Supplementary file 2 — Supplementary file2 (DOCX 21 KB) [file 431_2021_4200_MOESM2_ESM.docx]

| Supplementary Table 2: Summary of available literature on PE and Eculizumab use in STEC-HUS | | | | |
| --- | --- | --- | --- | --- |
| Author | Year | Study Type | Population | Outcome |
| Monet-Didailler et al., | 2020 | Matched Cohort | 18 C with Ecu (10 CNS) vs 36 C no Ecu | Renal outcome: no different between groups at 1y. 4/10 CNS treated with Ecu had neurological sequelae vs 1/9 untreated. |
| Percheron et al., | 2018 | Cohort Study | 33 C with Ecu (28 CNS) | 19/28 (68%) patients favourable neurological outcome |
| Agbas et al., | 2018 | Cohort (control) | 32 C (8 CNS)  9 Ecu | Complete neurological recovery in all patients, no difference in renal outcome |
| Loos et al., | 2017 | Cohort Study | 72 C (23 CNS) 11Ecu | Comparable outcomes at 3 years |
| Pape et al., | 2015 | Case Series | 11 C (11 CNS)  11 Ecu | No seizures after Ecu in 8 patients |
| Delmas et al., | 2014 | Cohort Study | 8 A & 1 C (3 CNS) 3 PE & 9 Ecu | Improvement in neurological status, favourable outcome in all |
| Gitiaux et al., | 2013 | Cohort Study | 7 C (CNS) 7 Ecu | Good neurological outcome in 5/7 children . Mortality 2/7 |
| Kielstein et al., | 2012 | Cohort Study | 491 A 193 Ecu & 334 PE | Significantly lower mortality with PE (3.7%) or PE/Ecu (2.6%) vs. supportive care (10.5%) |
| Menne et al., | 2012 | Case-Control | 298 A (37 CNS) 67 Ecu & 251 PE | No short term benefit reported |
| Loos et al., | 2012 | Cohort Study | 90 C (23 CNS) 1 Ecu & 17 PE | Overall outcome good, but no significant difference vs those with basic supportive care |
| Lapeyraque et al., | 2011 | Case Series | 3C (3 CNS) 3 Ecu | Improvement in neurological status within 24 hours |
| Colic et al., | 2011 | Cohort | 5 A (5 CNS) 5 PE | Complete recovery in all patients |
| Nathanson et al., | 2010 | Cohort Study | 25 C 25 PE | 5/25 (20%) no neurological sequelae |
| Dundas et al., | 1996 | Cohort | 22 A (7 CNS) 16 PE | Mortality 31% (PE) vs 91% (older cohort) |
| Gianviti et al., | 1993 | Case Control | 33 C 11 PE | Renal recovery statistically equivalent but better in PE (82% vs. 55%) at 1 year |
| Abbreviations: A, adults; C, children; Ecu, Eculizumab CNS, neurological involvement; PE, Plasma Exchange; yr,year | | | | |
