# Supplementary material for: Neurological involvement in children with hemolytic uremic syndrome
Source: Eur J Pediatr. 2021 Aug 10;181(2):501–12. doi: 10.1007/s00431-021-04200-1 (PMC8821508; doi:10.1007/s00431-021-04200-1)
Supplement: Supplementary file 3 — Supplementary file3 (DOCX 18 KB) [file 431_2021_4200_MOESM3_ESM.docx]

**Supplementary Table 3: Application of HUS early prediction score**

|  | **Number** | **﻿≤ 13**  **N (%)** | **>14**  **N (%)** | ***P-*value** |  |
| --- | --- | --- | --- | --- | --- |
|  |  |  |  |  |  |
| **Total group** | 202 | 99 (49) | 103 (51) | 0.902 |  |
| Neurological group | 22 | 11 (50) | 11 (50) | 0.902 |  |
| Non-neurological group | 180 | 88 (49) | 92 (51) | 0.902 |  |
|  | | | | |  |
| Data Available | 178 | 88 (49) | 90 (51) | 0.760 |  |
| Lost | 5 | 0 (0) | 5 (100) | 0.027 |  |
| Regional | 18 | 10 (56) | 8 (44) | 0.545 |  |
| RIP | 1 | 1 (100) | 0 (0) | 0.304 |  |
| **Short Term outcomes** |  |  |  |  |  |
| Dialysis | 107 | 26 (24) | 81 (76) | <0.0001 |  |
| Eculizumab | 8 | 2 (25) | 6 (75) | 0.170 |  |
| ICU admission | 48 | 20 (40) | 28 (60) | 0.192 |  |
| **Long-term outcome** |  |  |  |  |  |
| Complete recovery | 154 | 77 (50) | 77 (50) | 0.726 |  |
| Sequelae | 24 | 11 (46) | 13 (54) | 0.726 |  |
|  |  |  |  |  |  |
| Score calculated as per *﻿“A simple prognostic index for Shigatoxin-related hemolytic uremic syndrome at onset: data from the ItalKid-HUS network”, Ardissino et al., 2018*  **Formula:** Hb (g/dL) + [2 x Serum Creatinine (mg/dL) | | | | |  |
