# Supplementary figures and images for: Neurological involvement in children with hemolytic uremic syndrome
Source: Eur J Pediatr. 2021 Aug 10;181(2):501–12. doi: 10.1007/s00431-021-04200-1 (PMC8821508; doi:10.1007/s00431-021-04200-1)

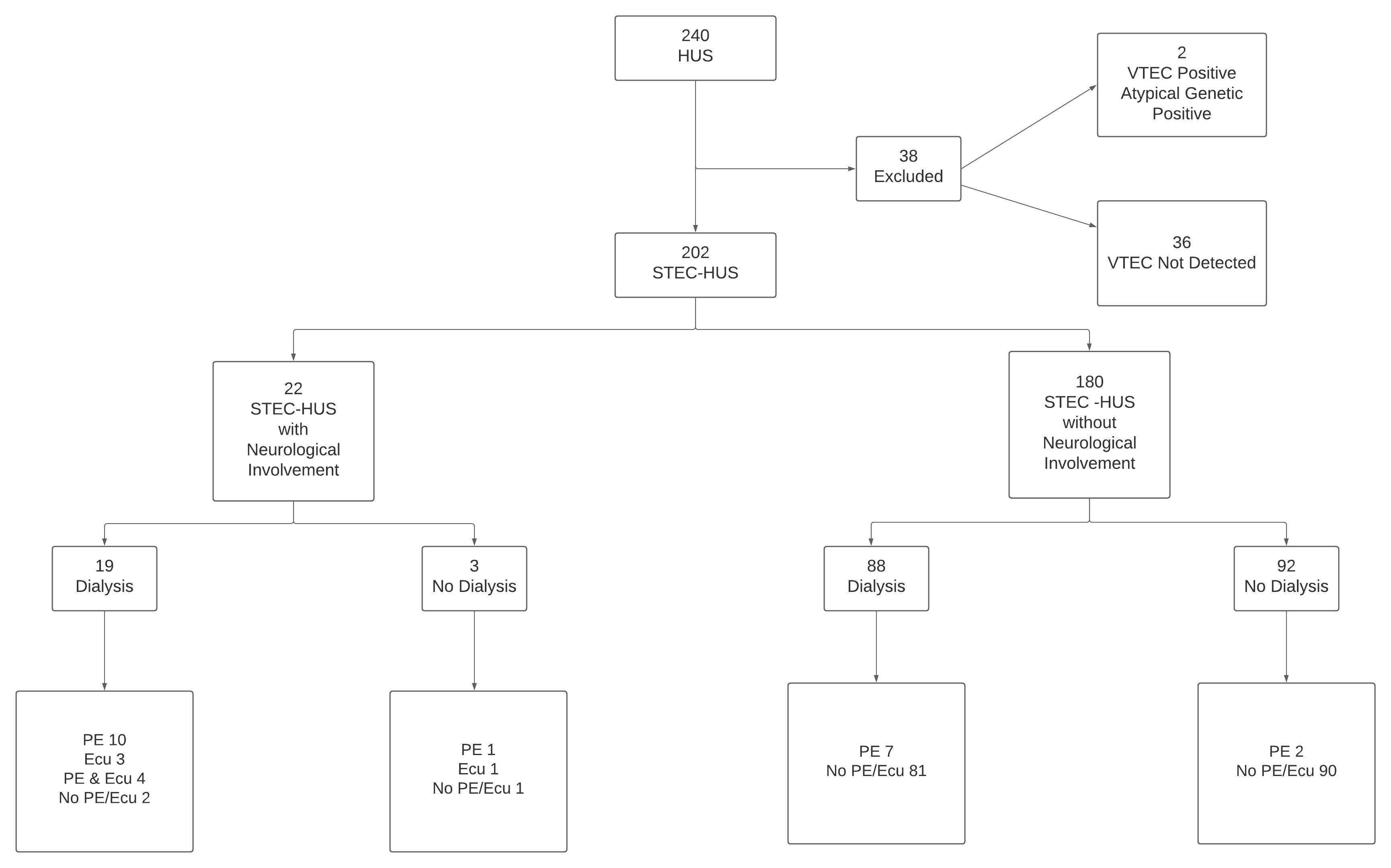

Supplement: Supplementary file 4 — Supplementary file4 (JPEG 149 KB) [file 431_2021_4200_MOESM4_ESM.jpeg]
